# Six-dimensional force/torque sensor for aerodynamic characteristic study of high-speed train with different wind angles under stationary tornado

**DOI:** 10.1371/journal.pone.0298401

**Published:** 2024-03-21

**Authors:** Mu Li, Yecheng Wu, Jiankun Gan, Bo Chai, Yuanzhao Zhang

**Affiliations:** 1 Beijing Jiaotong University, Beijing, China; 2 Beijing’s Key Laboratory of Structural Wind Engineering and Urban Wind Environment, School of Civil Engineering, Beijing Jiaotong University, Beijing, China; Jamia Millia Islamia, INDIA

## Abstract

Researchers conducted an investigation by tornado simulator to study the impact of wind angle on the aerodynamic characteristics of a reduced (1:150) high-speed train model using six-dimensional force/torque sensor. The reduced scale model size can match the relative size relationship between high-speed train and tornado vortex core in real condition. Results show that the wind angle affects the mean value and standard deviation of the force and moment coefficient of the high-speed train at the same radial position. The variations of the mean value and standard deviation of the pitching moment coefficient of the high-speed train carriage model at 60°and 90°are different from that at other wind angles. The variations of the mean value of the pitching moment coefficient of the high-speed train head model at 0°, 15°and 30°are different from that at other wind angles. The variations of the standard deviation of the pitching moment coefficient of the high-speed train head model at 60°,75°and 90°are different from that at other wind angles. This research will help the further study of the operation safety of high-speed train in the event of a tornado impacting a high-speed train network.

## Introduction

Tornadoes are known for their extremely destructive nature [1–3]. In China alone, the 2016 Yancheng tornado caused 99 fatalities and 846 injuries [4] while the Wenchang tornado in the same year claimed one life and injured 11 others [5]. The plain areas of China are prone to tornadoes. Since high-speed train (HST) network crossing the plains is highly distributed [6], the likelihood of HST being hit by tornadoes is increasing. In fact, two tornadoes in 2021 disrupted the regular operation of HST. On June 1, 2021, an EF2 tornado passed through the railway line twice in Shangzhi City, Heilongjiang Province, causing delays for six trains on Harbin-Mudanjiang High-Speed Railway. On July 21, 2021, a tornado in Qingyuan District, Baoding City, Hebei Province, led to the shutdown of HST service at Shijiazhuang Station. The Beijing-Guangzhou High-Speed Railway has implemented speed limits due to the threat of tornadoes. Japan has also experienced tornadoes hitting HST; on December 25, 2005, HST traveling on the Uetsu line was overturned by a tornado, resulting in five deaths and 32 injuries [7].

The aerodynamic characteristics of HST could be characterized by its force and moment coefficients, which are sensitive to wind. Therefore, studying the aerodynamic characteristics of HST with tornado conditions is of great practical significance. Crosswind has caused several HST and train accidents [8]. For example, the derailment accident of passenger train from Wulumuqi to Akesu was induced by strong crosswind in 2007, causing numerous casualties and significant property losses [9]. The effect of crosswind on HST is crucial for safety operation. Numerous researchers have investigated the aerodynamic characteristics of HST under crosswinds [10–17], which are influenced by conditions such as site type, wind angle, inclined barriers, windbreaks, and porous shelters. However, because tornado wind fields differ significantly from crosswind wind fields, these research results do not directly apply.

There are a few studies that have explored the aerodynamic characteristics of HST with tornado conditions specifically. Suzuki and Okura [18] conducted a pressure measurement test on a train model passing through a simulated tornado with a fixed wind angle, and the shape of train model is like high-speed train carriage (HSTC). They observed that the lateral force sign of the train changed when train traversed the tornado, and the lift force and yaw moment reached their maximum values near the tornado vortex core center. Obara et al. [19] conducted computational fluid dynamics (CFD) calculations to describe changes in the tornado wind field upon encountering a train; the CFD calculation results were similar to experimental results.

There are several studies on this subject have centered on HSTC, while little researches have also investigated the aerodynamics of high-speed train head (HSTH) with tornado conditions. Xu et al. [20] simulated the marshalling of HST with a fixed wind angle passing through tornado via the CFD method to find that the lateral force was minimal when the HSTH was near to the center of the tornado vortex core. The study of aerodynamic characteristic of HSTH within tornado condition is still limited and also not yet fully understood. The wind angles of HST are generally fixed for the purposes of such research, which affects the contact state between the HST and tornado vortex core, thus influencing the aerodynamic characteristics of the HST under tornado conditions. It is yet necessary to fully consider the impact of wind angles on the aerodynamic characteristics of HST with stationary tornado conditions.

To the best of our knowledge, the primary focus of previous research lies in HSTC, with a noticeable absence of experimental investigations centered on HSTH. The aerodynamic force and moment of HSTC via pressure measurement are obtained by integration of pressure data. The integration result depends on the measurement point locations on the HSTC model. In addition, the number of measurement points also affect the integration result. Thus, the integration of pressure measurement results may have errors. The six-dimensional force/torque sensor can measure the aerodynamic force and moment directly when HST model in the complex tornado wind field, and then the force and moment coefficient can be calculated. Though force sensor or force/torque sensor were used to study the aerodynamic forces of HST under boundary layer wind [12, 14], there is no relative research for aerodynamic characteristic study of HST under tornado using six-dimensional force/torque sensor based on author’s knowledge. The piezometer tube inside pressure measurement model prevents the reduction of the model size. The inside of the force test model is hollow and the force test model can be reduced to match the relative size relationship between high-speed train and tornado vortex core in real condition. The force test result approximately approaches the actual situation.

The primary goal of the present study was to investigate wind angles effect on the aerodynamic characteristics of the HSTH and HSTC under stationary tornado. The difference between aerodynamic shape of HSTH and HSTC is huge, and the aerodynamic characteristic of HSTH should be compared with that of HSTC. The tornado simulator we used was made by Beijing Jiaotong University and enables effective simulations of complex tornado wind fields. By using a small six-dimensional force/torque sensor (NANO17), we could directly measure the aerodynamic force and moment of a HST model in this simulated environment, and subsequently the aerodynamic characteristics of HST model can be obtained. Our findings may provide useful support for enhancing the operation safety of HST within tornado conditions in future.

## Experimental setup

### Tornado simulator

**Fig 1:**
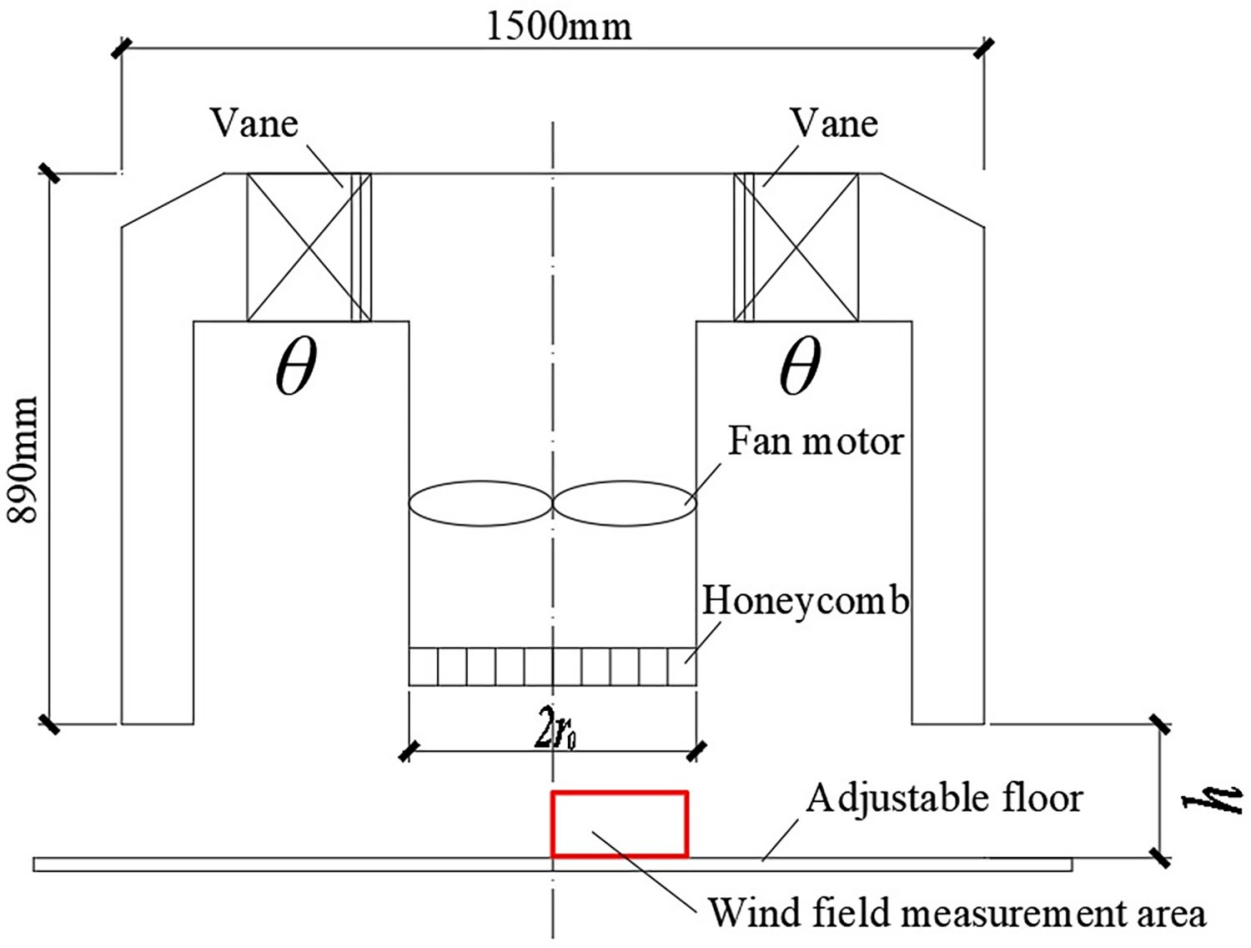
Beijing jiaotong university tornado simulator.

The tornado simulator was utilized in Beijing Jiaotong University to carry out a force test. The mechanism of this tornado simulator is similar to that of the “ISU” tornado simulator in Iowa State University [21]. The Beijing Jiaotong University tornado simulator is depicted in Fig 1. Its circular duct has a diameter and height of 1500 mm and 890 mm, respectively. The radius of the updraft hole (*r*_0_) is 250 mm.

Swirl ratio (*S*) is an important parameter that controls the simulated tornado wind field. For the Beijing Jiaotong University simulator, *S* can be defined by adjusting the degree (*θ*) of vane at the top. The range of *θ* is 0–60°. The definition of *S* is *S* = *tanθ*/2*a*, where *a* is the aspect ratio of the tornado, *a* = *h*/*r*_0_. We set *h* to 300 mm, *S* to 0.35, and the corresponding *θ* to 40°,

### Simulated tornado wind field

We used a TFI Cobra probe to measure the simulated tornado wind field and the sampling frequency is 1250 Hz. The wind velocity measurement area of the simulated tornado is shown in Fig 1. The wind velocity measurement area for height range (*z*) and radial distance (*r*) are 10–200 mm and 0–300 mm, respectively. The simulated tornado wind field is three-dimensional, comprised of tangential wind velocity, radial wind velocity, vertical wind velocity, and pressure drop components. Tangential wind velocity (*U*) and pressure drop (*P*) were the main components under investigation.

**Fig 2:**
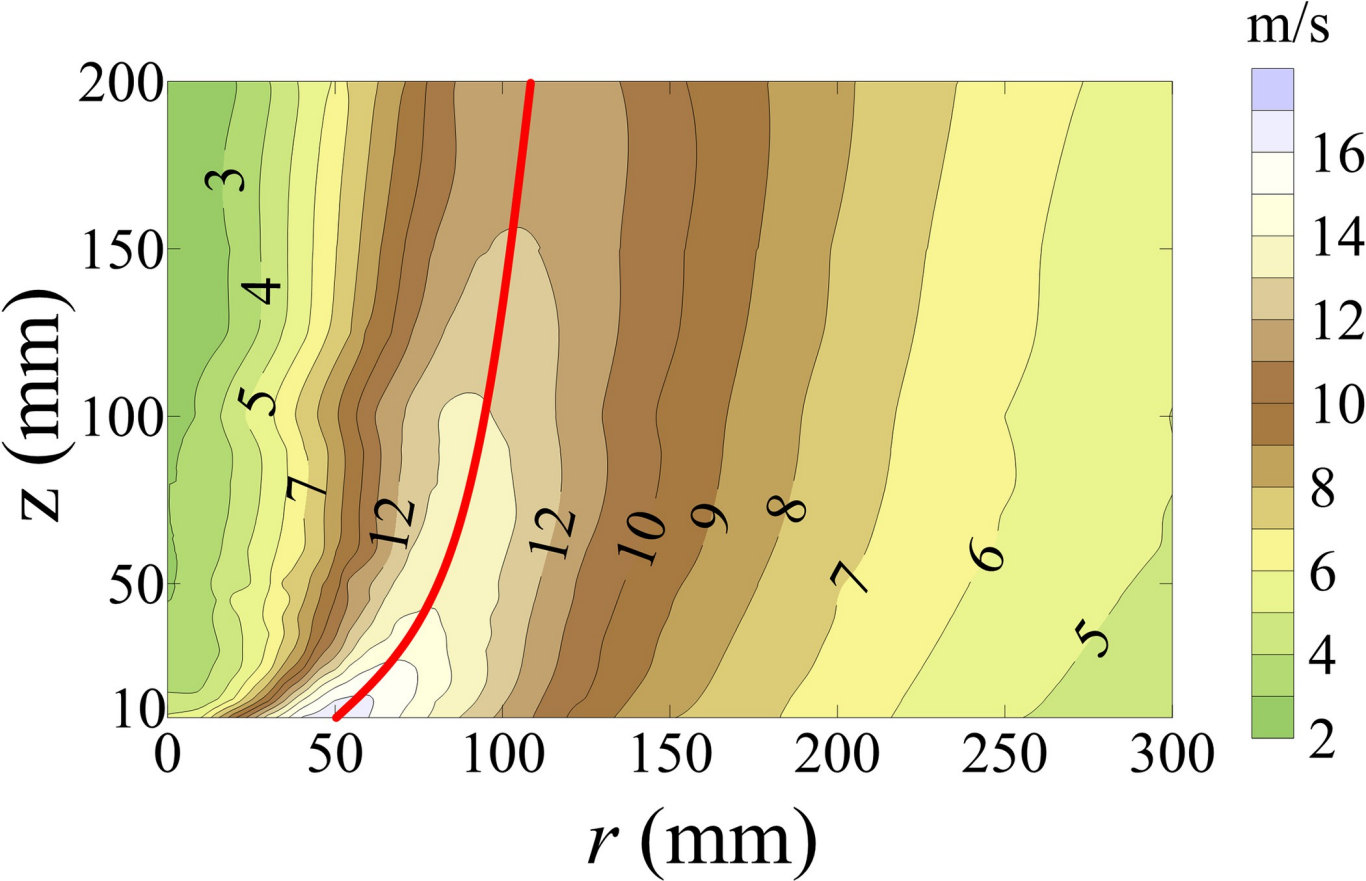
Contour map of *U*_*t*_.

Fig 2 shows the mean value of tangential wind velocity (*U*_*t*_). The radial position of the maximum *U*_*t*_ (*U*_*t*,*max*_) within the same wind field height is the tornado vortex core (*r*_*c*_). The red line in Fig 2 represents the *r*_*c*_ value along the entire wind field height. *U*_*t*,*max*_ gradually decreases as wind field height increases. As *r* increases at the same wind field height, *U*_*t*_ increases first to reach *U*_*t*,*max*_ at *r*_*c*_, then decreases. The horizontal gradient of *U*_*t*_ inside the region of vortex core is greater than that outside and at the region of vortex core.

**Fig 3:**
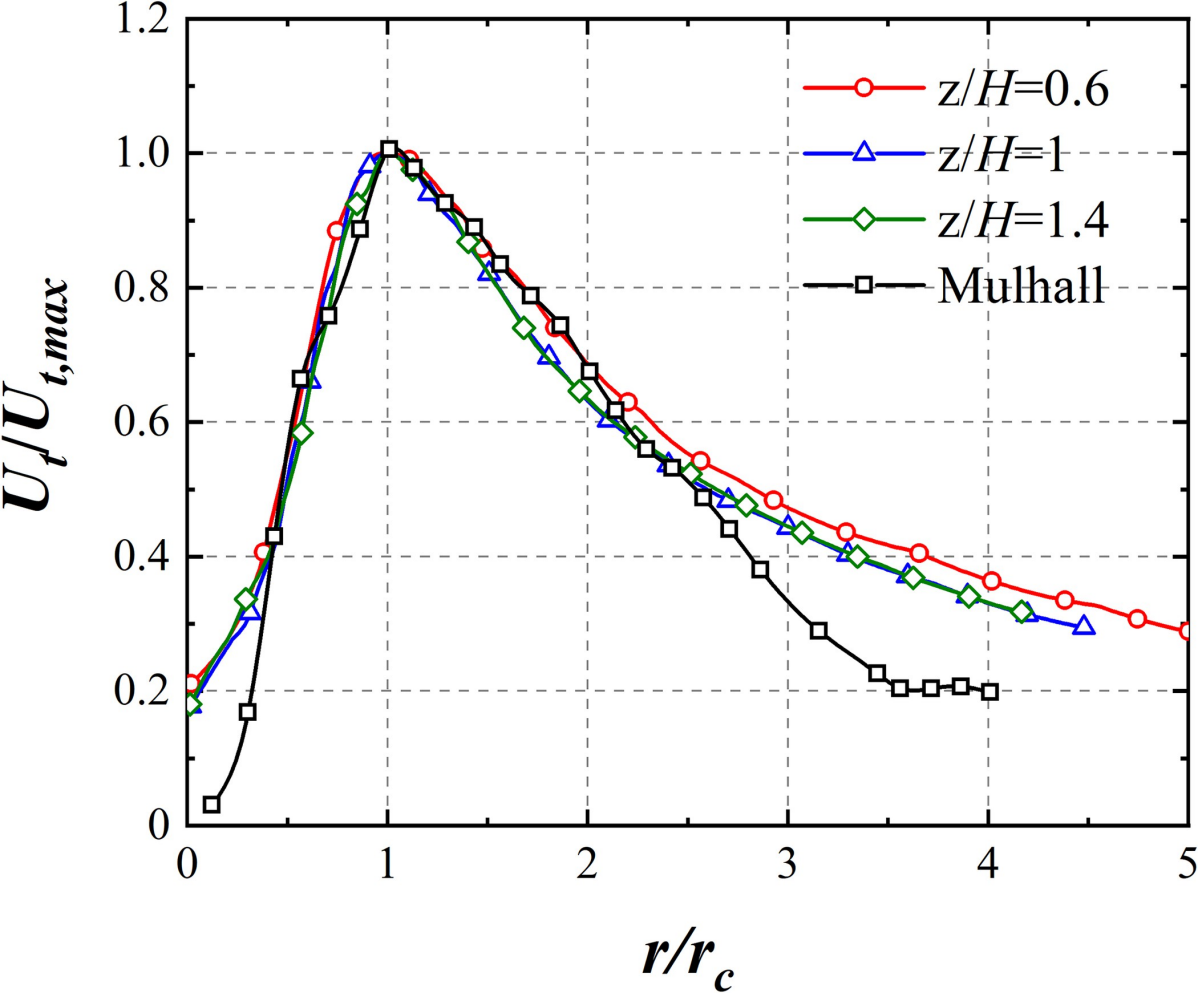
Radial distribution of *U*_*t*_.

The radial distribution of *U*_*t*_ at heights of 15 mm, 25 mm, and 35 mm is shown in Fig 3, where *H* represent the maximum height of HST model (Details regarding HST model are provided in Section HST model and force test), alongside a comparison with the field data of the Mullhall torando [22]. *U*_*t*_ and *r* are normalized by *U*_*t*,*max*_ and *r*_*c*_, respectively. The radial distribution of *U*_*t*_ for the simulated tornado aligns with that of Mullhall tornado.

**Fig 4:**
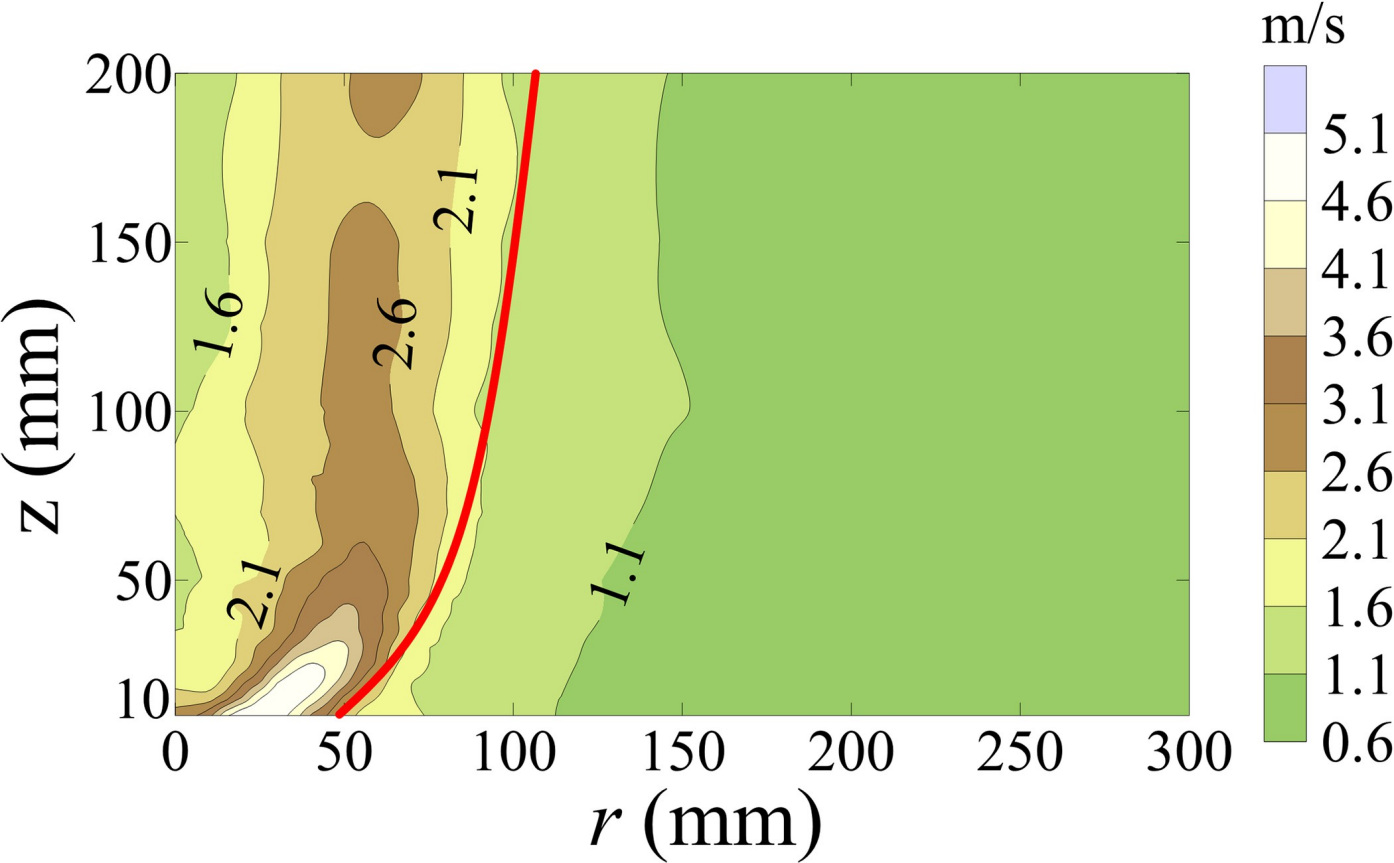
Contour maps of *U*_*std*_.

The contour maps of standard deviation of *U* (*U*_*std*_) is shown in Fig 4. The value of *U*_*std*_ inside *r*_*c*_ is greater than that outside *r*_*c*_, which indicates that the fluctuating component of tornado inside *r*_*c*_ is greater than that outside *r*_*c*_, shown in Fig 4.

**Fig 5:**
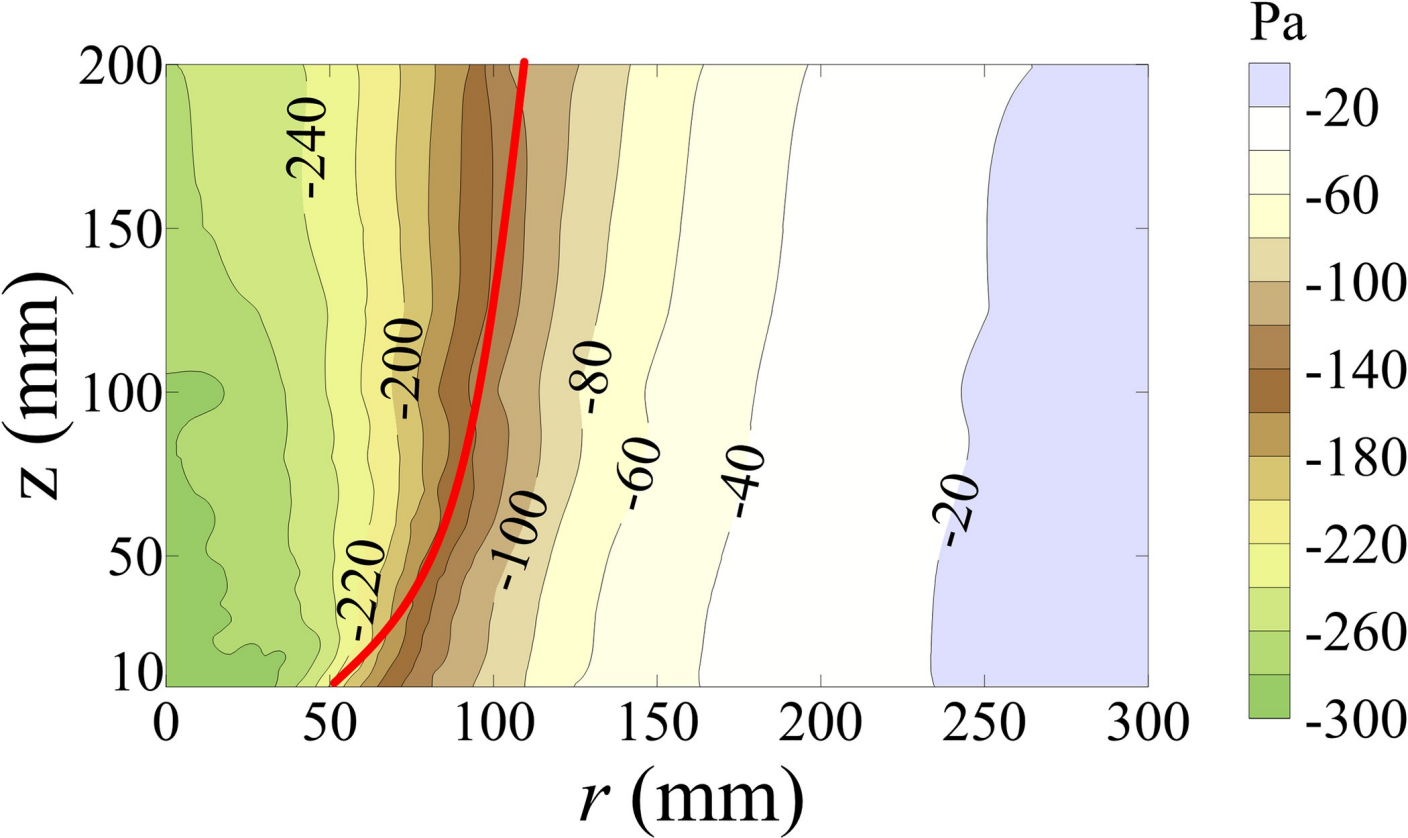
Contour map of *P*_*t*_.

The mean value of pressure drop (*P*_*t*_) is shown in Fig 5. At the same wind field height, |*P*_*t*_| increases as *r* decreases, which indicates that the suction of the tornado is largest at its vortex core center. The horizontal gradient of *P*_*t*_ is greater at the region of *r*_*c*_ than at other radial positions.

**Fig 6:**
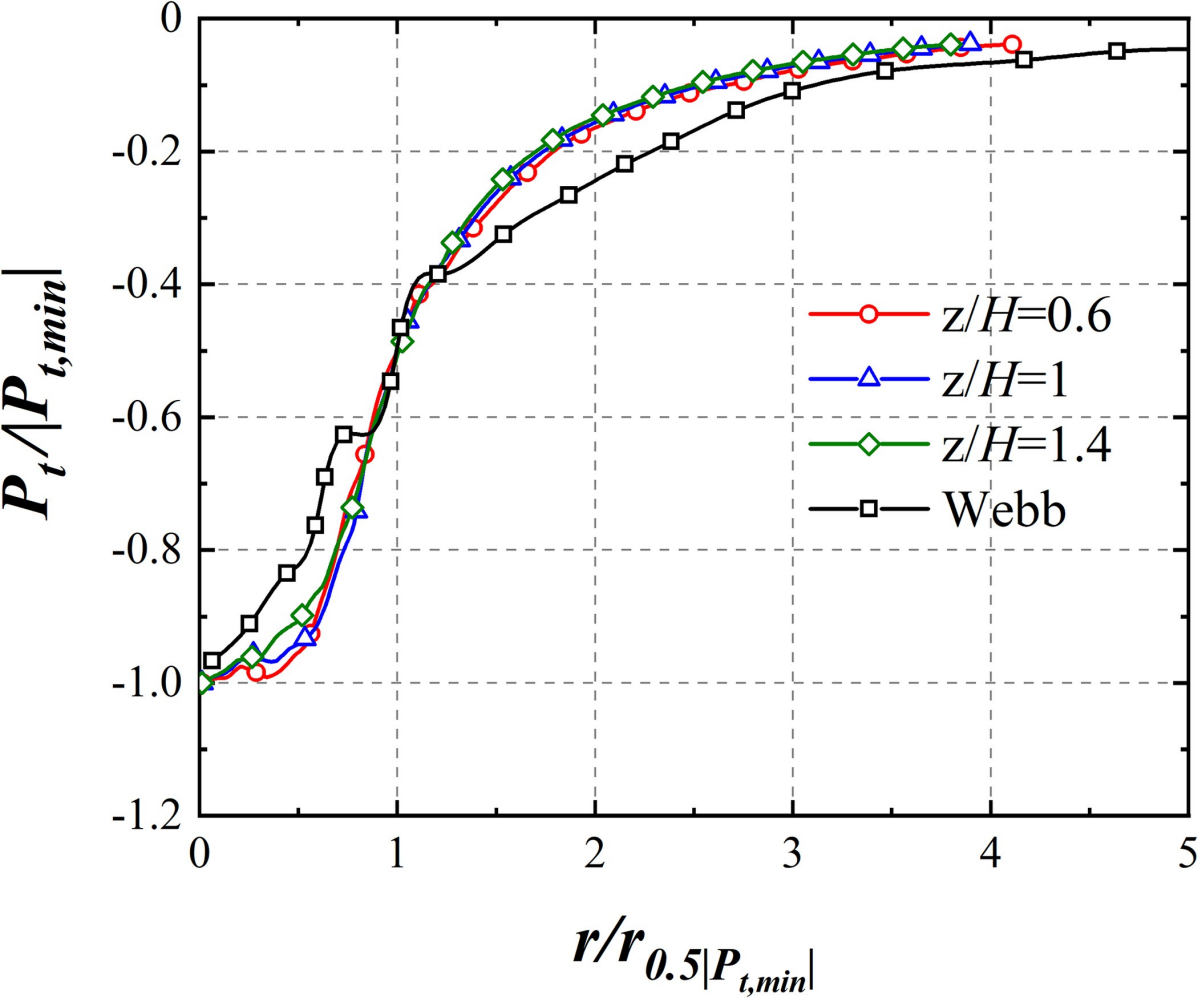
Radial distribution of *P*_*t*_.

The radial distribution of *P*_*t*_ at heights of 15 mm, 25 mm, and 35 mm is shown in Fig 6, alongside a comparison with field data for the Webb tornado [23]. *P*_*t*_ is normalized by absolute value of minimum *P*_*t*_ (|*P*_*t*,*min*_|) within the same wind field height and *r* is normalized by radial position ($r_{0.5|P_{t,min}|}$) where half of |*P*_*t*,*min*_|(0.5|*P*_*t*,*min*_|) occurs. The radial distribution of *P*_*t*_ for the simulated tornado aligns with that of Webb tornado.

**Fig 7:**
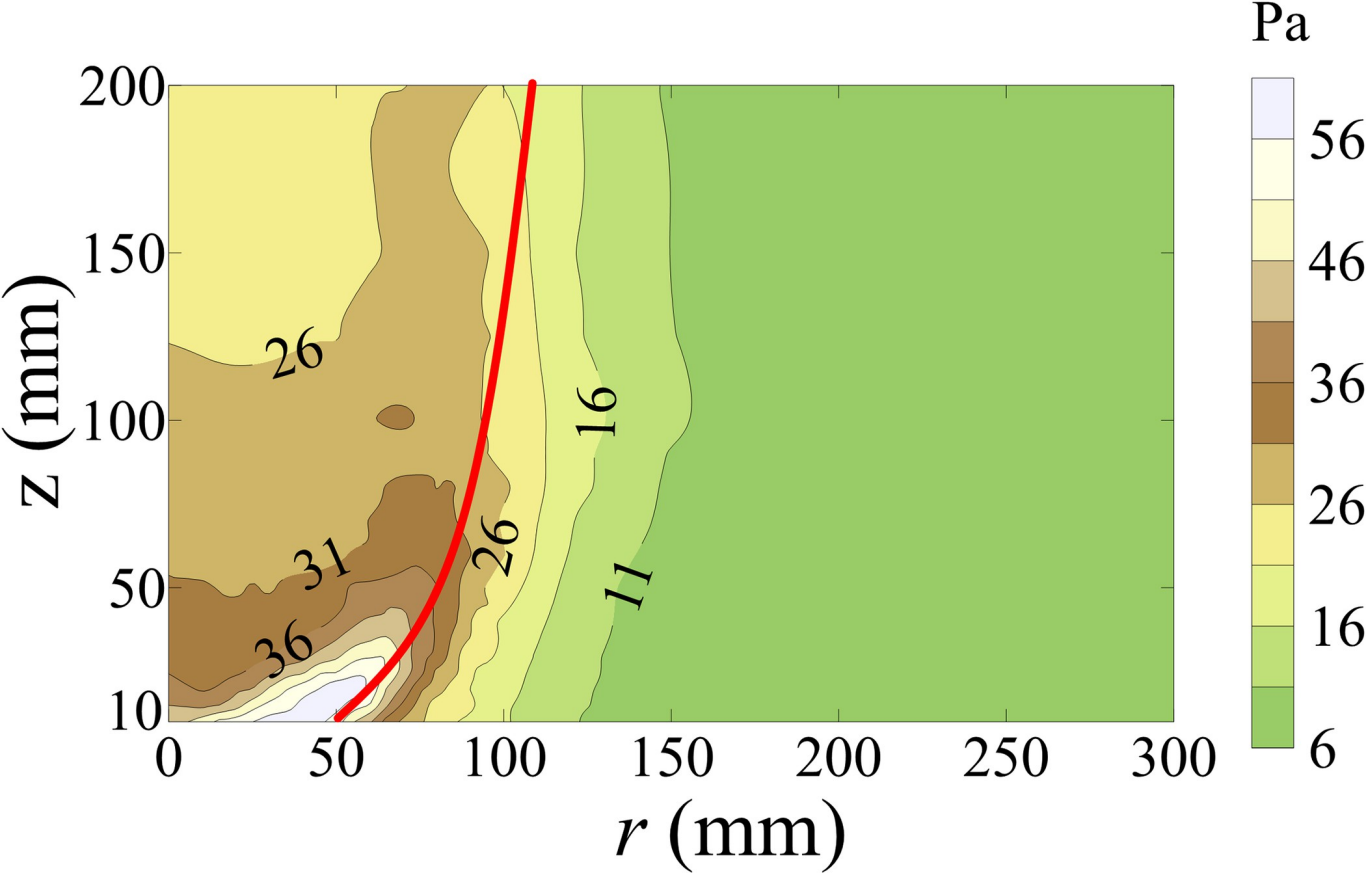
Contour maps of *P*_*std*_.

Contour maps for the standard deviation of *P* (*P*_*std*_) is shown in Fig 7. The value of *P*_*std*_ inside *r*_*c*_ is greater than that outside *r*_*c*_, which also indicates that the fluctuating component of tornado inside *r*_*c*_ is greater than that outside *r*_*c*_.

### HST model and force test

A high frequency force/torque sensor can be used to directly measure the aerodynamic force and moment of a HST, which represent its aerodynamic characteristics. Due to the limited size of the tornado simulator, tornado vortex core size is also limited. The force test model size can be reduced to match the relative size relationship between the HST model and the simulated tornado vortex core, ensuring that the force test results are realistic. The NANO17 (SI-50-0.5), a six- dimensional force/torque sensor made by ATI, was used in this study to measure the aerodynamic force and moment of a CRH380A model prototype, which is the most common type of HST operating in Chinese railway. The aerodynamic shape of CRH380A was designed by CRRC QINGDAO SIFANG CO,.LTD. The max test speed of CRH380A is 423.7km/h on Shanghai-Hangzhou High-speed Railway. By 2020, the total number of CRH380A is 448. This HST model with CRH380A prototype was selected to ensure that this research approximates real-world practical operation conditions of HST during tornado events. The diameter and height of NANO17 are 17 mm and 14.5 mm, respectively. NANO17 is currently the smallest size force/torque sensor produced by ATI. The sensor measurement spans of X-axis force, Y-axis force and Z-axis force are 0~50N, 0~50N and 0~70N, respectively. The sensor measurement spans of X-axis torque, Y-axis torque and Z-axis torque are 0~0.5Nm. The sensor measurement spans ensure that the sensor will not overload during the connection process with the high-speed train model because overload is irreversible damage for force/torque sensor. The resolutions of three-axis force and three-axis torque are 1/80N and 1/0.016Nm, respectively, and therefore the accuracy of the force test can be guaranteed.

**Fig 8:**
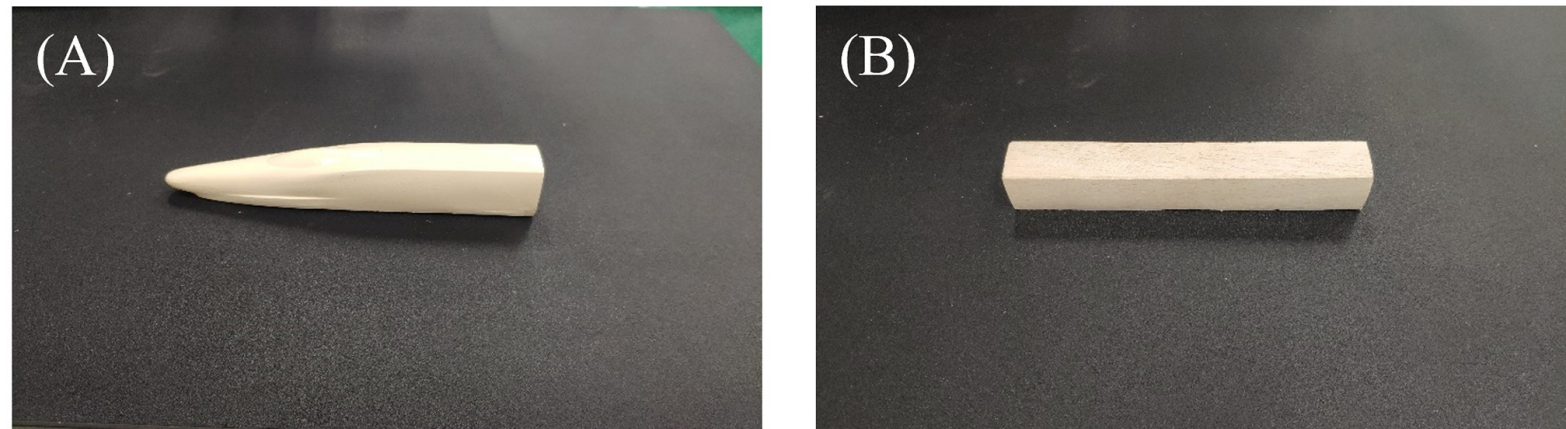
Photos of HST models. (A) HSTH model. (B) HSTC model.

The aerodynamic shapes of the HSTH and HSTC differ. Researchers conducted force tests of the HSTH and HSTC separately. The train model was reduced to a scale of 1:150, with a maximum width(*W*) and maximum height(*H*) of 25 mm; the pantograph, bogie, and wheel, among other components, were not included in the model because they are too small to display on HST model (Fig 8). Because HST operates in marshalling mode, the force test of a single train should include the influence of adjacent carriages, which is referred to here as the “interference model”. The cross-sectional shape of the interference model is like that of the HSTC model, and the interference model length is 50 mm.

**Fig 9:**
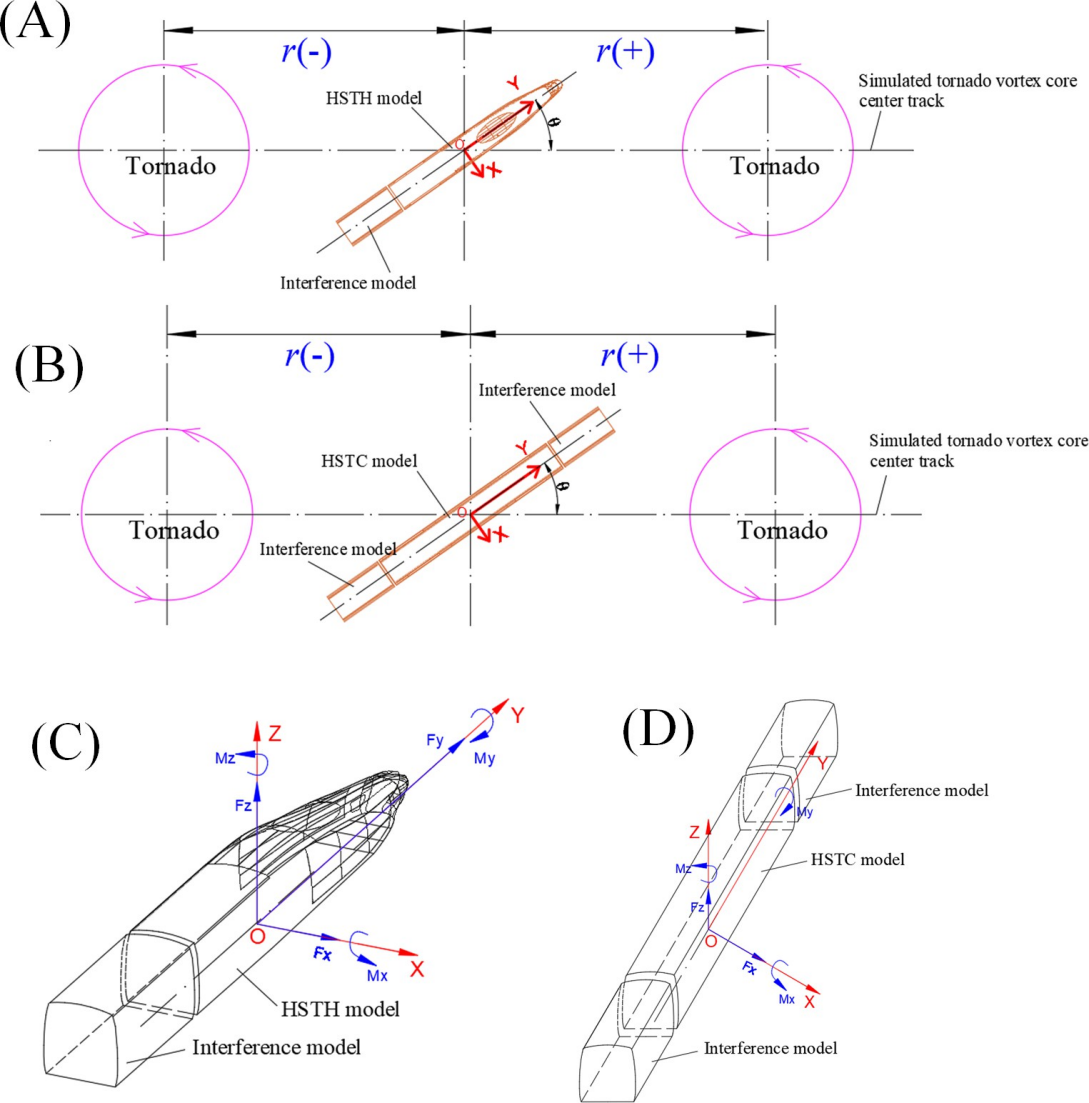
Layouts(top view) of HST models. (A) HSTH model. (B) HSTC model; Reference system of HST models. (C) HSTH model. (D) HSTC model.

Interference models were placed at both ends of the HSTC model, and at non-nose end of the HSTH model. The longitudinal axis of the interference model is consistent with the longitudinal axis of the HST model. To ensure the force test accuracy, the mass of HST model was kept as small as possible. HST models were fabricated from wood with hollow interiors, and interference models were also made by wood. There was only a 1–2 mm interval between the interference model and the HST model. The layout of the model is shown in Fig 9A and 9B.

During the test, the position of the HST model remained fixed as the tornado simulator was moved to different radial positions (*r*) relative to the model while the simulated tornado vortex core center track passed through the bottom origin of the HST model (O) which is at the middle of the lower surface of HST model, as shown in Fig 9. O is also the origin of the reference system of force-moment measurement, and the reference systems of force-moment measurement for HST model are shown in Fig 9C and 9D.*r* is the distance between O and the simulated tornado vortex core center. *r* is negative if the simulated tornado vortex core center is located on the left side of the model, and vice versa, see Fig 9A and 9B. Researchers tested 23 values of *r* for the high-speed train model including ±300 mm(±12*W*), ±250 mm(±10*W*), ±200 mm(±8*W*), ±160 mm(±6.4*W*), ±140 mm(±5.6*W*), ±120 mm(±4.8*W*), ±100 mm(±4*W*), ±80 mm(±3.2*W*), ±60 mm(±2.4*W*), ±40 mm(±1.6*W*), ±20 mm(±0.8*W*), and 0 mm. The sampling frequency of sensor was 312 Hz and the sampling time for each *r* was 120 s. The sampling frequency of this research will ensure the reflection of aerodynamic characteristic of HST.

The wind angle (*θ*) of the HST is a key parameter affecting its aerodynamic characteristics. The definition of *θ* is also shown in Fig 9A and 9B. There are six wind angles for the HSTC model: 0°, 15°, 30°, 45°, 60°, and 90°. Compared with the HSTC, the HSTH shape is irregular. Accordingly, researchers tested seven wind angles for the HSTH: 0°, 15°, 30°, 45°, 60°, 75°, and 90°. The total test conditions for the HSTH and HSTC were 7×23 = 161 and 6×23 = 138, respectively.

**Fig 10:**
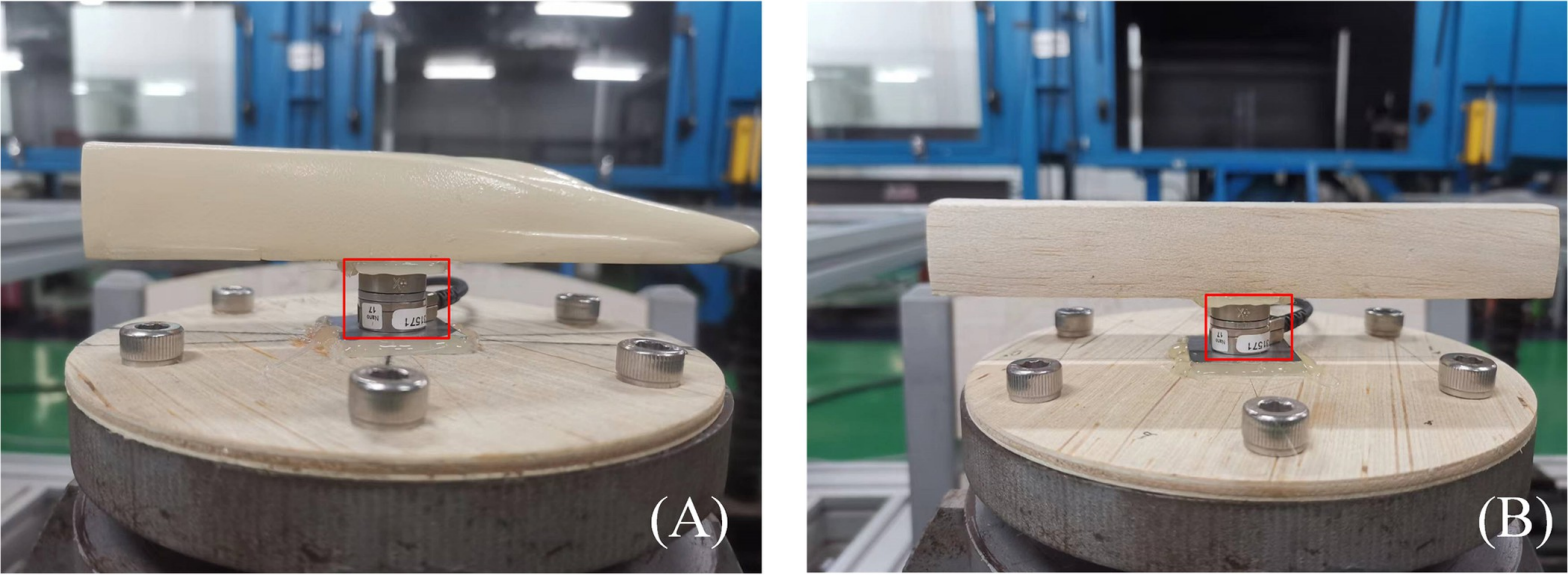
The integration between NANO17 and HST models. (A) HSTH model. (B) HSTC model.

The force/torque sensor used is located at O of the HST model, where it could simultaneously measure the aerodynamic force and moment according to lateral force (*F*_*x*_), drag force (*F*_*y*_), lift force (*F*_*z*_), pitching moment (*M*_*x*_), rolling moment (*M*_*y*_), and yaw moment (*M*_*z*_), as shown in Fig 9C and 9D. The integration between NANO17 and the HST model is shown in Fig 10, where red frames indicate the force/torque sensor. As there were interference models at both ends of the HSTC model, the drag force of the HSTC model was not considered (Fig 9D). The aerodynamic force coefficients and moment coefficients can be calculated according to the aerodynamic force and moment measurements as follows:

$$C_{Fx}=\frac{F_{x}}{0.5\rho V^{2}\cdot LH}$$ (1)

$$C_{Fy}=\frac{F_{y}}{0.5\rho V^{2}\cdot WH}$$ (2)

$$C_{Fz}=\frac{F_{z}}{0.5\rho V^{2}\cdot LW}$$ (3)

$$C_{Mx}=\frac{M_{x}}{0.5\rho V^{2}\cdot L^{2}W}$$ (4)

$$C_{My}=\frac{M_{y}}{0.5\rho V^{2}\cdot LH^{2}}$$ (5)

$$C_{Mz}=\frac{M_{z}}{0.5\rho V^{2}\cdot L^{2}H}$$ (6)

where *C*_*Fx*_, *C*_*Fy*_, *C*_*Fz*_, *C*_*Mx*_, *C*_*My*_, and *C*_*Mz*_ represent lateral force coefficient, drag force coefficient, lift force coefficient, pitching moment coefficient, rolling moment coefficient, and yaw moment coefficient, respectively. *C*_*Fy*_ is not included in the HSTC model. *L* is length of HST model; *W* and *H* are the maximum width and maximum height of the cross-section of the HST model, respectively. *ρ* is air density and *V* is the maximum tangential wind velocity at *H*.

**Fig 11:**
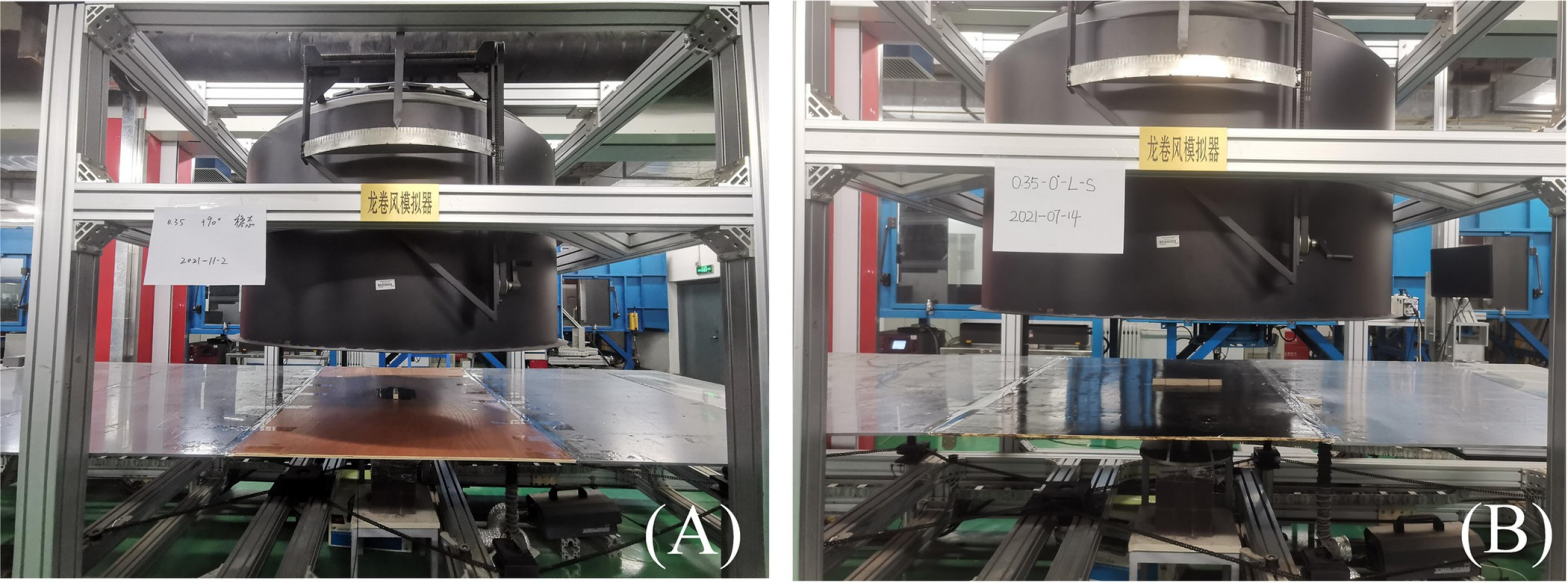
Force test photos of HST models. (A) HSTH model. (B) HSTC model.

For the convenience, *C*_*Fx*,*c*_, *C*_*Fz*,_, *C*_*Mx*,*c*_, *C*_*My*,*c*_, and *C*_*Mz*,*c*_ are used here to represent lateral force coefficient, lift force coefficient, pitching moment coefficient, rolling moment coefficient, and yaw moment coefficient of the HSTC model, respectively. *C*_*Fx*,*h*_, *C*_*Fy*,*h*_, *C*_*Fz*,*h*_, *C*_*Mx*,*h*_, *C*_*My*,*h*_, and *C*_*Mz*,*h*_ represent the lateral force coefficient, drag force coefficient, lift force coefficient, pitching moment coefficient, rolling moment coefficient, and yaw moment coefficient of the HSTH, respectively. Photos of the HST force test under stationary tornado conditions are shown in Fig 11, where tornado simulator is on the moveable crane, and the radial position of HST model is fixed. Throughout the experiment, tornado simulator was adjusted to different radial positions to finish force test.

## Aerodynamic characteristics of HSTC model

**Fig 12:**
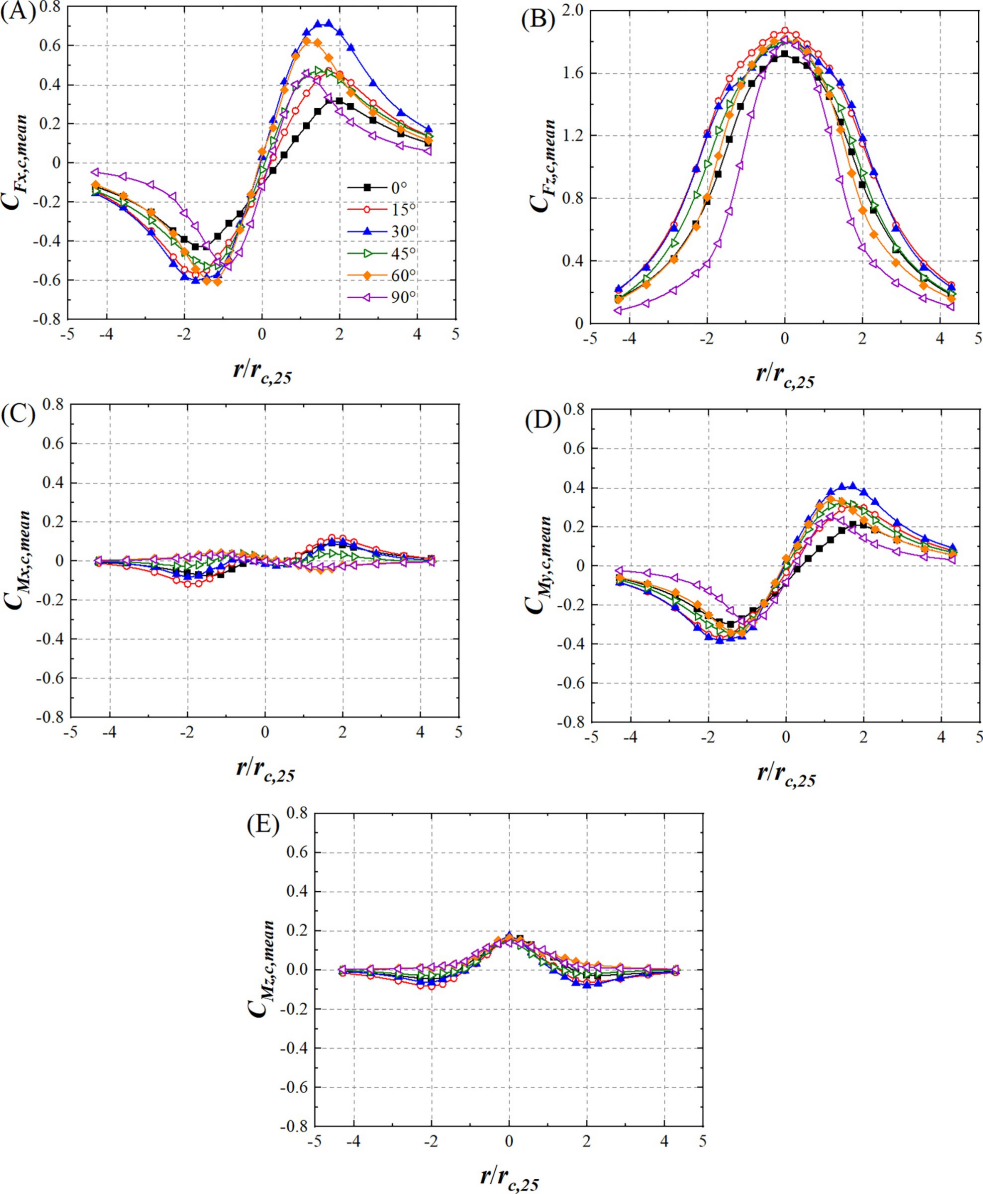
The variations in mean value of force and moment coefficient of HSTC model. (A) *C*_*Fx*,*c*,*mean*_, (B) *C*_*Fz*,*c*,*mean*_, (C) *C*_*Mx*,*c*,*mean*_, (D) *C*_*My*,*c*,*mean*_ and (E) *C*_*Mz*,*c*,*mean*_.(*r*_*c*,25_ is 70mm).

Fig 12 shows the variations in lateral force coefficient mean value (*C*_*Fx*,*c*,*mean*_), lift force coefficient mean value (*C*_*Fz*,*c*,*mean*_), pitching moment coefficient mean value (*C*_*Mx*,*c*,*mean*_), rolling moment coefficient mean value (*C*_*My*,*c*,*mean*_), and yaw moment coefficient mean value (*C*_*Mz*,*c*,*mean*_) of the HSTC model for all wind angles with respect to *r*/*r*_*c*,25_. *r* is normalized by *r*_*c*,25_, where *r*_*c*,25_ is 70 mm which is the tornado vortex core radius at the height of 25 mm (Fig 2). Negative *r*/*r*_*c*,25_ and positive *r*/*r*_*c*,25_ values state that the simulated tornado vortex core center is at the left and right side of HST model, respectively. Wind angle has no effect on the variations of *C*_*Fx*,*c*,*mean*_, *C*_*Fz*,*c*,*mean*_, or *C*_*My*,*c*,*mean*_. |*C*_*Fx*,*c*,*mean*_| and |*C*_*My*,*c*,*mean*_| at all wind angles first increase and then decrease as *r*/*r*_*c*,25_ increases, as shown in Fig 12A and 12D, indicating that rolling moment is mainly caused by lateral force. *C*_*Fz*,*c*,*mean*_ decreases at all wind angles as |*r*/*r*_*c*,25_| increases, as shown in Fig 12 B. The variations in *C*_*Mx*,*c*,*mean*_ and *C*_*Mz*,*c*,*mean*_ at *θ* = 60° and *θ* = 90° differ from those at other wind angles, as shown in Fig 12C and 12E, implying that wind angles affect the contact state between the HSTC model and the tornado vortex core. The variation in *C*_*Fx*,*c*,*mean*_ and *C*_*Fz*,*c*,*mean*_ when *θ* = 0° in this research is similar to Suzuki and Okura’s finding [18]. The size of tornado vortex core is limited, and the tornado vortex core is 70mm at the wind field height of 25mm(Fig 2) which is the height of HST model. The radial position range of this force test is from -300mm to +300mm which is greater than 70mm. *U*_*t*_ decreases drastically outside the region of tornado vortex core, and the absolute value of *P*_*t*_ starts to decrease from tornado vortex core center. Thus, radial position is an important factor influencing the mean value of force coefficient and moment coefficient of HST model, and this influence is responsible for the marked fluctuations that can be observed in Fig 12.

*C*_*Fx*,*c*,*mean*_ values at all wind angles reach nearly 0 when *r*/*r*_*c*,25_ = 0, as shown in Fig 12A. The pressure drop has little effect on the lateral force of the HSTC model when *r*/*r*_*c*,25_ = 0. The tangential airflow directions of the tornado acting on both ends of the HSTC model oppose each other and the tangential airflow intensity is approximately equal at either end, which make for a lateral force of approximately 0 for all wind angles at *r*/*r*_*c*,25_ = 0. As |*r*/*r*_*c*,25_| increases, the pressure drop begins to affect the lateral force of HSTC, and the tangential airflow of both ends does not oppose each other. Thus, |*C*_*Fx*,*c*,*mean*_| begins to increase to the maximum value. The size of tornado vortex core is limited, and |*C*_*Fx*,*c*,*mean*_| begins to decreases after |*C*_*Fx*,*c*,*mean*_| reaches the maximum value as |*r*/*r*_*c*,25_| increases. *C*_*Fx*,*c*,*mean*_ values differ under different wind angles obtains different value as |*r*/*r*_*c*,25_| increases. This is because the contact state between the HSTC model and the tornado vortex core differs under different wind angles at the same radial position, which alters the tangential airflow and pressure drop of the tornado acting on the model. *C*_*Fz*,*c*,*mean*_ and *C*_*Mz*,*c*,*mean*_ reach their maximum values at all wind angles when *r*/*r*_*c*,25_ = 0, indicating that lift force and swirling effect are strongest at *r*/*r*_*c*,25_ = 0, respectively. *C*_*Fz*,*c*,*mean*_ gradually decreases with the increase in |*r*/*r*_*c*,25_| because the effect of pressuredrop on lift force gradually diminishes as HSTC model moves away from tornado vortex core. As |*r*/*r*_*c*,25_| increases, the value of *C*_*Fz*,*c*,*mean*_ at *θ* = 90° becomes smaller than that at other wind angles, indicating that HSTC model first disengages from the tornado vortex core at *θ* = 90° with increase in |*r*/*r*_*c*,25_|. Accordingly, the influence of tornado pressure drop on the lift force of the HSTC model rapidly weakens at *θ* = 90°.

**Fig 13:**
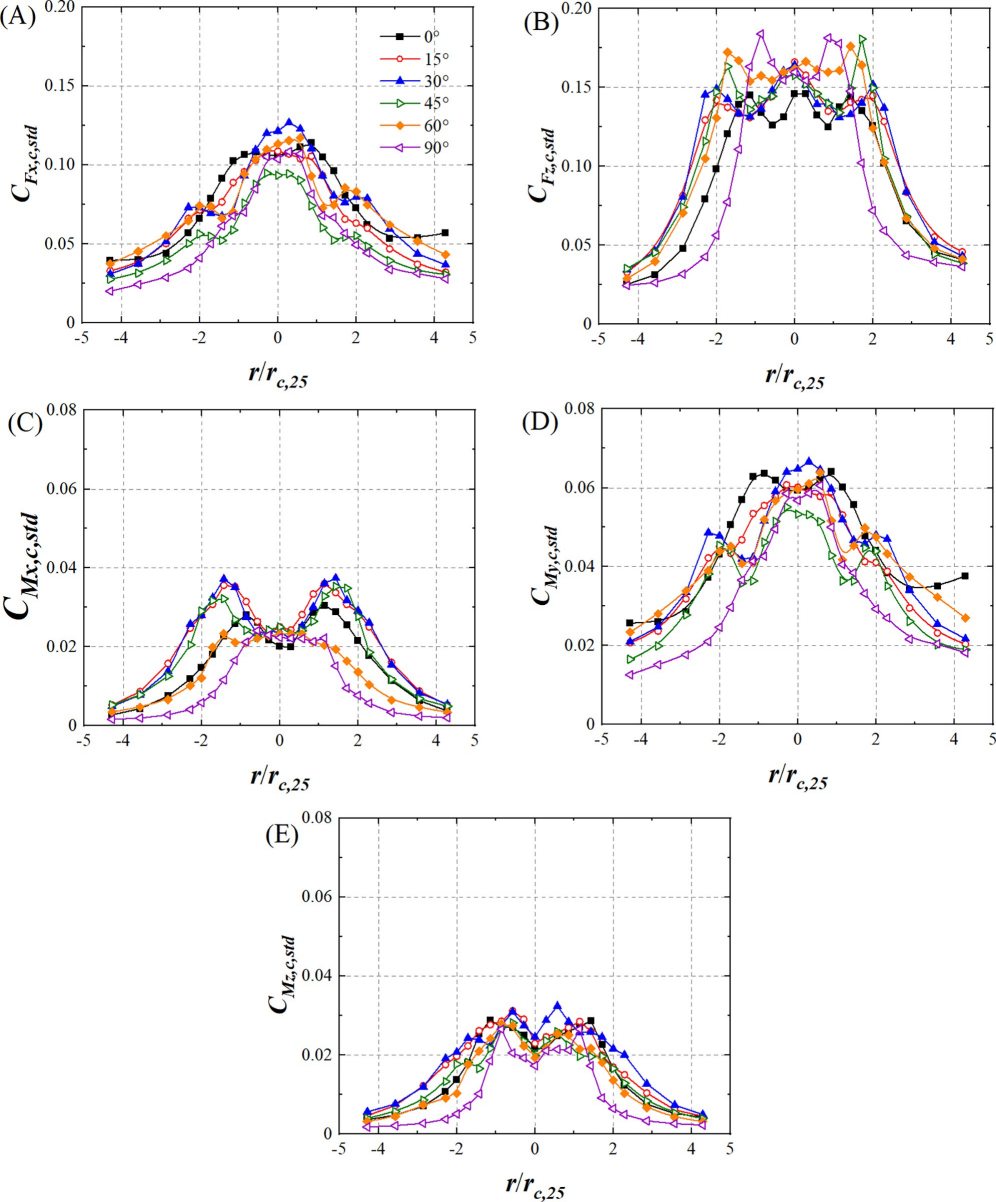
The variations in standard deviation of force and moment coefficient of HSTC model. (A) *C*_*Fx*,*c*,*std*_, (B) *C*_*Fz*,*c*,*std*_, (C) *C*_*Mx*,*c*,*std*_, (D) *C*_*My*,*c*,*std*_ and (E) *C*_*Mz*,*c*,*std*_.(*r*_*c*,25_ is 70mm).

Fig 13 shows the variations in the lateral force coefficient standard deviation (*C*_*Fx*,*c*,*std*_), lift force coefficient standard deviation (*C*_*Fz*,*c*,*std*_), pitching moment coefficient standard deviation (*C*_*Mx*,*c*,*std*_), rolling moment coefficient standard deviation (*C*_*My*,*c*,*std*_), and yaw moment coefficient standard deviation (*C*_*Mz*,*c*,*std*_) of the HSTC model for all wind angles with respect to *r*/*r*_*c*,25_. The variations in *C*_*Fx*,*c*,*std*_ and *C*_*My*,*c*,*std*_ at all wind angles trend downward as |*r*/*r*_*c*,25_| increases, while the radial distribution of *C*_*Fz*,*c*,*std*_ at all wind angles remains basically axisymmetric along the longitudinal axis. *C*_*Mx*,*c*,*std*_ shows an M-shaped variation trend when *θ*<60° (Fig 13C) while *C*_*Mz*,*c*,*std*_ shows an M-shaped variation trend at all wind angles (Fig 13E). The standard deviation of the force coefficient and moment coefficient differs within different wind angles at the same radial position, implying that wind angle affects the contact state between the HSTC model and the vortex core of the transient tornado wind field. The standard deviation of the force coefficient and moment coefficient in the region of |*r*/*r*_*c*,25_|<2 is also greater than that of |*r*/*r*_*c*,25_|>2, because the fluctuating component of the simulated tornado wind field is larger in the region of *r*/*r*_*c*,25_<2 than in *r*/*r*_*c*,25_>2. Further, *U*_*std*_ and *P*_*std*_ are larger in the region of *r*/*r*_*c*,25_<2 than in *r*/*r*_*c*,25_>2, as shown in Figs 4 and 7.

## Aerodynamic characteristics of HSTH model

**Fig 14:**
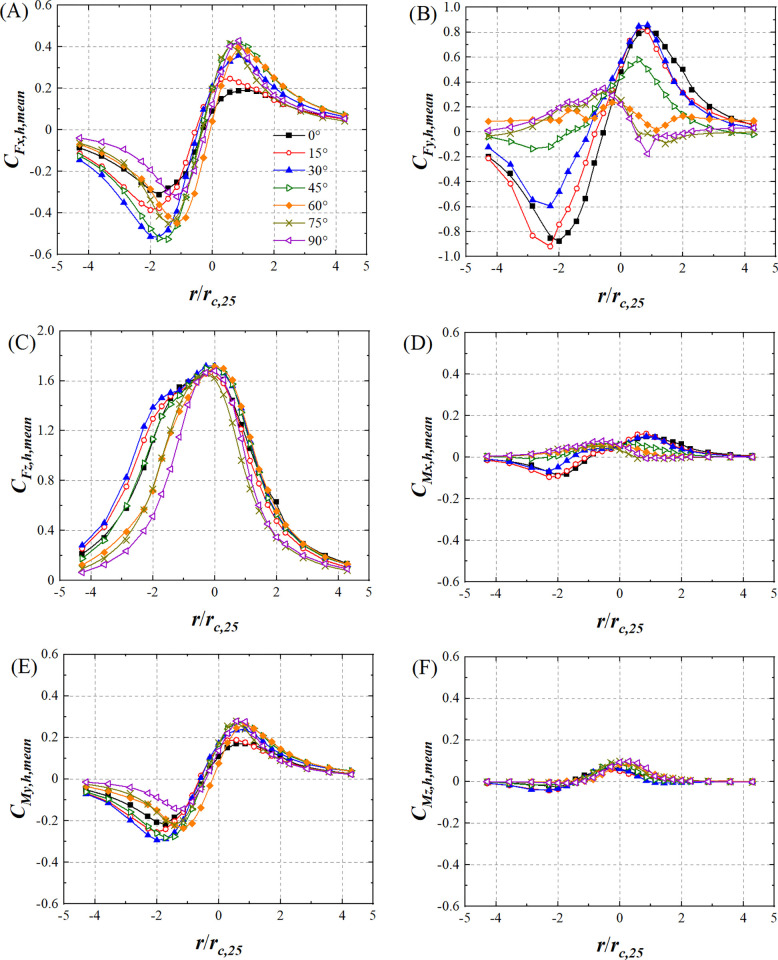
The variations in mean value of force and moment coefficient of HSTH model. (A) *C*_*Fx*,*h*,*mean*_, (B) *C*_*Fy*,*h*,*mean*_, (C) *C*_*Fz*,*h*,*mean*_, (D) *C*_*Mx*,*h*,*mean*_, (E) *C*_*My*,*h*,*mean*_ and (F) *C*_*Mz*,*h*,*mean*_ (*r*_*c*,25_ is 70mm).

Fig 14 shows the variations in lateral force coefficient mean value (*C*_*Fx*,*h*,*mean*_), drag force coefficient mean value (*C*_*Fy*,*h*,*mean*_), lift force coefficient mean value (*C*_*Fz*,*h*,*mean*_), pitching moment coefficient mean value (*C*_*Mx*,*h*,*mean*_), rolling moment coefficient mean value (*C*_*My*,*h*,*mean*_), and yaw moment coefficient mean value (*C*_*Mz*,*h*,*mean*_) for the HSTH model at all wind angles with respect to *r*/*r*_*c*,25_. Wind angle has no effect on the *C*_*Fx*,*h*,*mean*_ and *C*_*My*,*h*,*mean*_ variations. At all wind angles, |*C*_*Fx*,*h*,*mean*_| and |*C*_*My*,*h*,*mean*_| first increase and then decrease as |*r*/*r*_*c*,25_| increases (Fig 14A and 14E). The variations in *C*_*Fy*,*h*,*mean*_ differ at *θ*≤45° and *θ*>45° (Fig 14B), implying that wind angle affects *C*_*Fy*,*h*,*mean*_. *C*_*Fz*,*h*,*mean*_ decreases at all wind angles as |*r*/*r*_*c*,25_| increases (Fig 14C). Unlike *C*_*Fz*,*c*,*mean*_, the radial distribution of *C*_*Fz*,*h*,*mean*_ when *θ*≤45° does not have axial symmetry because HSTH model is asymmetric and therefore the lift force generated by pressuredrop when tornado vortex core located on left side and right side of HSTH model is different. When *θ* is greater than 45°, HSTH model is close to the vertical line, and effect of model asymmetry to the lift force is weakened. The *C*_*Mx*,*h*,*mean*_ variations at *θ*<45° differ from those at *θ*≥45°. This phenomenon can be attributed to the contact state between the HSTH model at different wind angles and the tornado vortex core, as well as the asymmetry of the HSTH model. Similar variation in *C*_*Fz*,*h*,*mean*_ when *θ* = 0° was observed by R Xu et al [20].

At all wind angles, *C*_*Fx*,*h*,*mean*_ moves away from 0 when *r*/*r*_*c*,25_ = 0 (Fig 14A) because the tangential airflow intensity acting on the both ends of HSTH model is not equivalent due to the asymmetry of HSTH model. *C*_*My*,*h*,*mean*_ is mainly affected by *C*_*Fx*,*h*,*mean*_. Thus, *C*_*My*,*h*,*mean*_ at all wind angles also moves away from 0 when *r*/*r*_*c*,25_ = 0 (Fig 14E). The *C*_*M*x,*h*,*mean*_ value at all wind angles is not 0 when *r*/*r*_*c*,25_ = 0that is different from *C*_*M*x,*h*,*mean*_, as shown in Fig 14D, which is also attributable to the asymmetry of the HSTH model.

**Fig 15:**
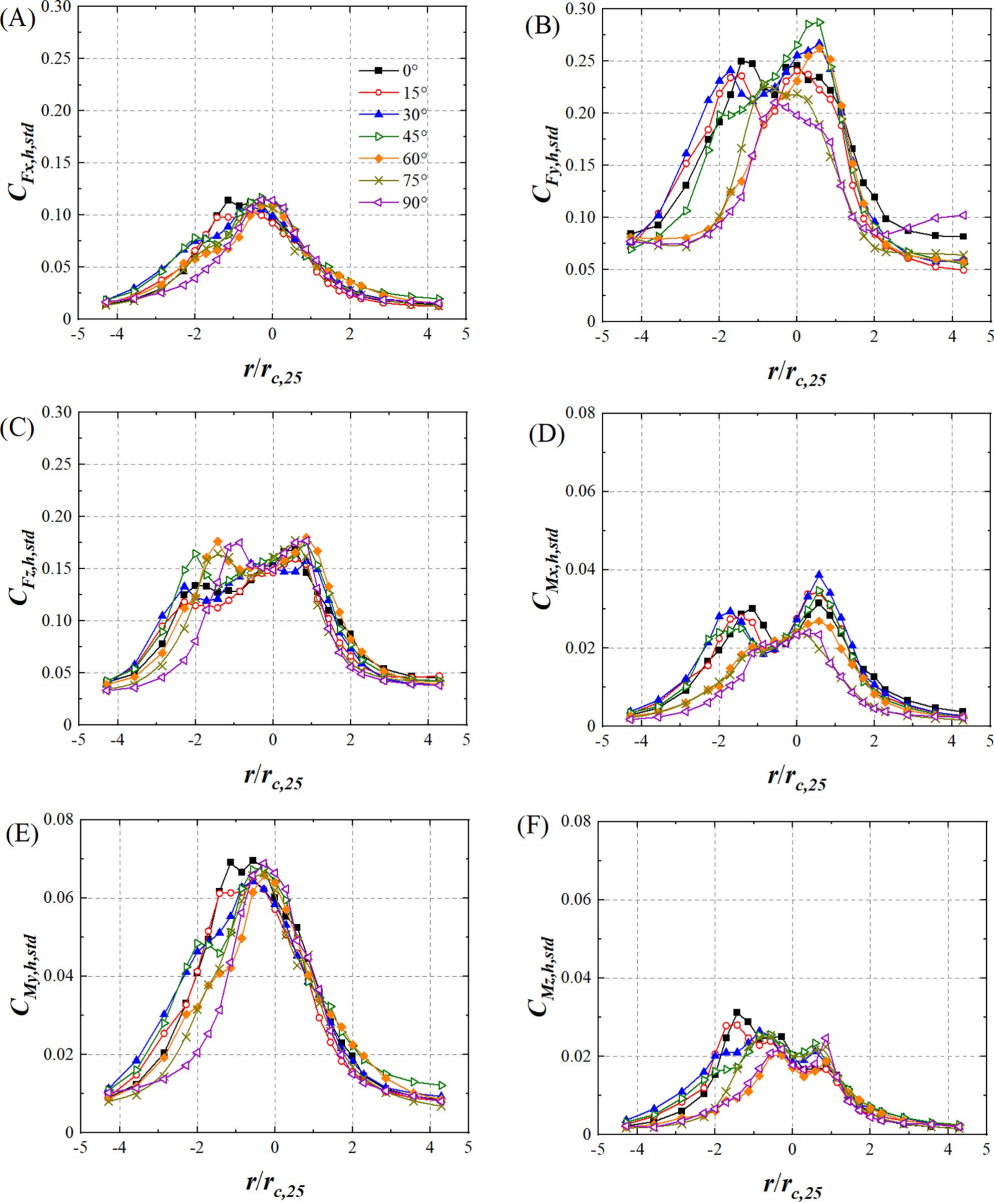
The variations in standard deviation of force and moment coefficient of HSTH model. (A) *C*_*Fx*,*h*,*std*_, (B) *C*_*Fy*,*h*,*std*_, (C) *C*_*Fz*,*h*,*std*_, (D) *C*_*Mx*,*h*,*std*_, (E) *C*_*My*,*h*,*std*_ and (F) *C*_*Mz*,*h*,*std*_. (*r*_*c*,25_ is 70mm).

Fig 15 shows the variations in lateral force coefficient standard deviation (*C*_*Fx*,*h*,*std*_), drag force coefficient standard deviation (*C*_*Fy*,*h*,*std*_), lift force coefficient standard deviation (*C*_*Fz*,*h*,*std*_), pitching moment coefficient standard deviation (*C*_*Mx*,*h*,*std*_), rolling moment coefficient standard deviation (*C*_*My*,*h*,*std*_), and yaw moment coefficient standard deviation (*C*_*Mz*,*h*,*std*_) of the HSTH model for all wind angles with respect to *r*/*r*_*c*,25_. At all wind angles, *C*_*Fx*,*h*,*std*_ and *C*_*My*,*h*,*std*_ increase first and then decrease as *r*/*r*_*c*,25_ increases. The radial distributions of *C*_*Fx*,*h*,*std*_, *C*_*Fz*,*h*,*std*_, *C*_*My*,*h*,*std*_, and *C*_*Mz*,*h*,*std*_ are roughly consistent at all wind angles when *r*/*r*_*c*,25_>0, but are no longer consistent when *r*/*r*_*c*,25_<0 which is caused by the asymmetry of the HSTH model. *C*_*Mx*,*h*,*std*_ shows an M-shaped variation trend when *θ*≤45°. *C*_*Mx*,*h*,*std*_ increases first and then decreases as *r*/*r*_*c*,25_ increases when *θ*>45°, as shown in Fig 15D. The standard deviation of the force coefficient and moment coefficient of the HSTH in the region of *r*/*r*_*c*,25_<2 is larger than that of *r*/*r*_*c*,25_>2, which is similar to the HSTC model.

## Conclusion

In this study, the aerodynamic characteristics of HSTH and HSTC models were studied by NANO17 force/torque sensor in Beijing Jiaotong University. Researchers also investigated the effects of various wind angles on the HST under stationary tornado conditions. Our findings can be summarized as follows.

Wind angle influences the aerodynamic characteristic of HST under stationary tornado. For HSTC model, wind angle does not affect the variations in *C*_*Fx*,*c*,*mean*_, *C*_*Fz*,*c*,*mean*_, and *C*_*My*,*c*,*mean*_. However, wind angle does affect value of *C*_*Fx*,*c*,*mean*_, *C*_*Fz*,*c*,*mean*_, and *C*_*My*,*c*,*mean*_ at the same radial position. The variation in *C*_*Mx*,*c*,*mean*_ and *C*_*Mz*,*c*,*mean*_ at 60°and 90°wind angles is different from that at other wind angles. For HSTH model, wind angle does not affect the variations in *C*_*Fx*,*h*,*mean*_ and *C*_*My*,*h*,*mean*_, but does affect the values of *C*_*Fx*,*h*,*mean*_ and *C*_*My*,*h*,*mean*_ at the same radial position. *C*_*Mx*,*h*,*mean*_ varies in a different pattern when wind angle is equal to or greater than 45° compared to wind angles smaller than 45°. The radial distribution of *C*_*Fz*,*h*,*mean*_ does not show axial symmetry when wind angle is less than or equal to 45°, though this is not the case for HSTC model. These findings may contribute to a more comprehensive understanding the aerodynamic characteristic of HST with different wind angles under tornado, particularly for HST located at or near the region of tornado vortex core. The aerodynamic characteristic of HST appears to be dependent on the radial position which is different from boundary layer wind. The variation in force and moment coefficient of HST can be used to establish the early tornado-warning system of HST.

The standard deviation of the force coefficient and moment coefficient of the HST model in the region of |*r*/*r*_*c*,25_|<2 is larger than that in the region of |*r*/*r*_*c*,25_|>2. The variation in *C*_*Mx*,*c*,*std*_ and *C*_*Mx*,*h*,*std*_ when wind angles is smaller than 60°varies in a different pattern compared to other wind angles. In the region of *r*/*r*_*c*,25_<2, the fluctuating tornado wind field will have a higher intensity on HST compared with other radial position range which may influence the stability and passenger comfort of HST.

Based on the force and moment coefficients identified in this study, the aerodynamic force and moment of HST under tornado can be obtained. More detailed analyses of the operational safety of HST under tornado may be conducted in future studies.
